# Supplementary material for: High-level expression of the monomeric SARS-CoV-2 S protein RBD 320-537 in stably transfected CHO cells by the EEF1A1-based plasmid vector
Source: PLoS One. 2021 Feb 2;16(2):e0242890. doi: 10.1371/journal.pone.0242890 (PMC7853477; doi:10.1371/journal.pone.0242890)
Supplement: S1 Raw images — (DOCX) [file pone.0242890.s005.docx]

Gels and blots raw images list

For manuscripts submitted on or after July 1 2019, authors must provide the original, uncropped and minimally adjusted images supporting all blot and gel results reported in an article’s figures and supporting information files. Whilst it is not necessary to provide original images at time of initial submission, we will require these files during the peer review process or before a manuscript can be accepted.

Please follow these instructions when preparing and submitting blot/gel data files:

- Please create a single PDF file that contains all the original blot and gel images contained in the manuscript’s main figures and supplemental figures. We recommend using image software (e.g. Gimp, Photoshop) to compile and annotate the original images and then exporting/saving as a tiff file with LZW compression. You could also use a PDF program to build a single PDF compiled from multiple annotated jpeg/tiff image files. We do not recommend compiling the images in Powerpoint as resolution can be lost.
- The file should be named ‘S1_raw_images’ and uploaded as a Supporting Information file or deposited at a suitable [publicly-available data repository](https://journals.plos.org/plosone/s/recommended-repositories), with the dataset identifier (DOI or other form of persistent identifier) provided in the [Data Availability Statement](https://journals.plos.org/plosone/s/data-availability).
- Authors should label each raw blot or gel image to clearly annotate the loading order, identity of experimental samples, method used to capture the image, and to specify which figure panel was generated from that original image. Molecular weight markers should be included or indicated on the raw image, and any lanes not included in the final figure should be marked with an “X” above the lane label on the original blot/gel image. All labeling and annotation should be performed without obscuring any data or background bands.
- Please note, there is a 20 MB maximum file size. If your PDF size is larger, please use a suitable repository or discuss with the journal staff.


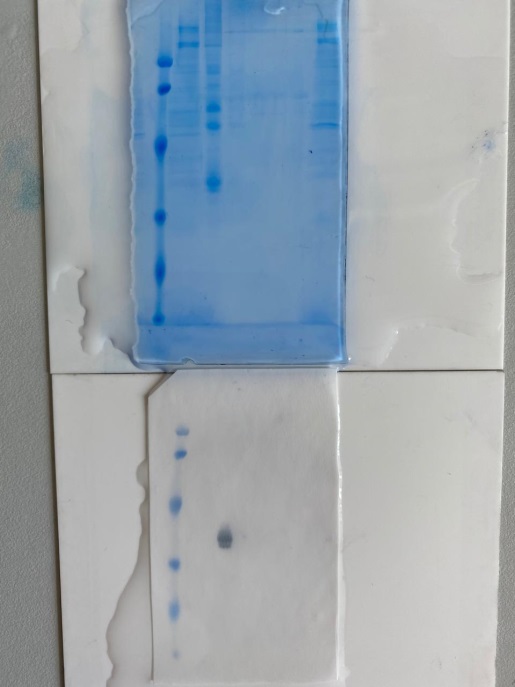


Fig_1B_Fig_1C - WhatsApp Image 2020-06-12 at 15.14.24.jpeg

11 june 2020
RBDv1 SDS-PAGE 12.5% and blot, purification on iminodiacetic acid (IDA)
lane 1: Prestained Protein Molecular Weight Marker #26612, 5 uL
lane 2: non-binding fraction, 5 uL concentrate
lane 3: 50 mM imidazole elution, 5 uL concentrate
lane 4: 50 mM imidazole elution (tail), 5 uL concentrate
lane 5: 100 mM imidazole elution, 5 uL concentrate
lane 6: 250 mM imidazole elution, 5 uL concentrate
lane 7: Na-EDTA elution, 5 uL concentrate
lane 8: harvested medium, 5 uL concentrate

All samples are 20x concentrates through Vivaspin PES membrane 10000 MWCO (Sartorius, UK, VS0102), SDS-PAGE 12.5% in reducing conditions (50 mM DTT)


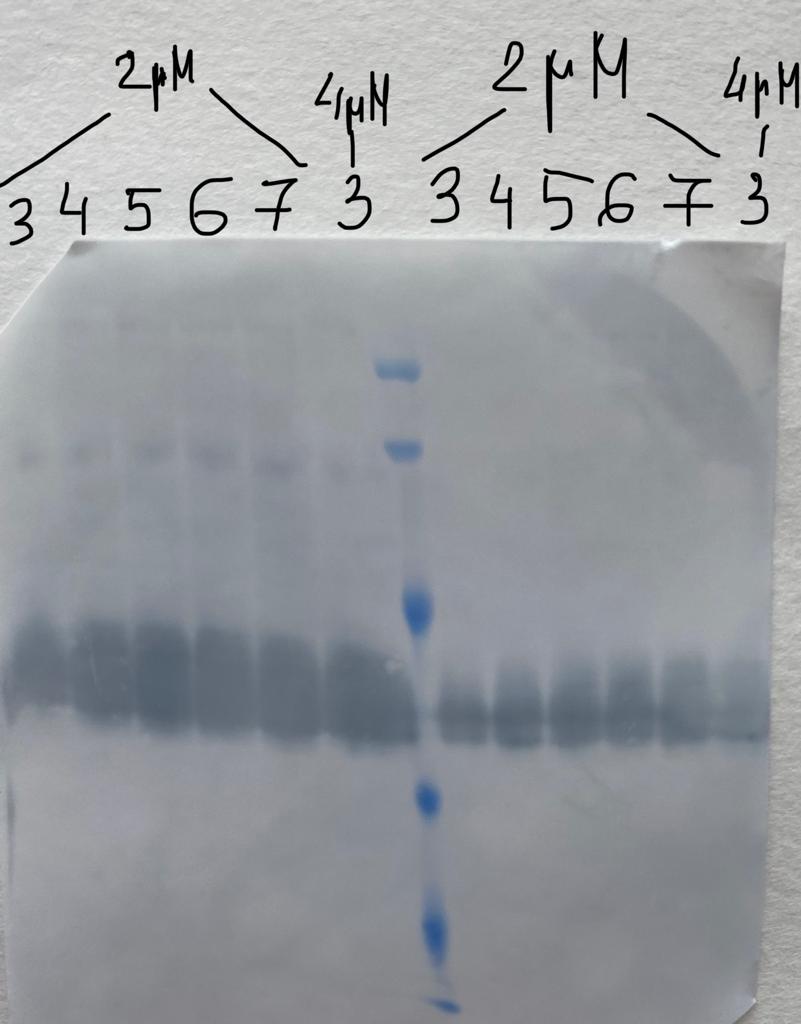


**X X X X X X**

Fig_1D - d585c824-6486-486b-87c3-39b542531e7b.jpg

23 july 2020
RBDv1 SDS-PAGE 12.5%
lanes 1-5: 2 uM RBDv1 culture, 10 uL concentrate from days 3 to 7 (batch process)
lane 6: 4 uM RBDv1 culture, 10 uL concentrate from day 3 (batch process)
lane 7: Prestained Protein Molecular Weight Marker #26612 5 uL
lanes 8-12: 2 uM RBDv1 culture, 1 uL concentrate from days 3 to 7 (batch process) – non-relevant data
lane 13: 4 uM RBDv1 culture, 1 uL concentrate from day 3 (batch process) – non-relevant data

All samples are 20x concentrates through Vivaspin PES membrane 10000 MWCO (Sartorius, UK, VS0102), SDS-PAGE 12.5% in reducing conditions (50 mM DTT)


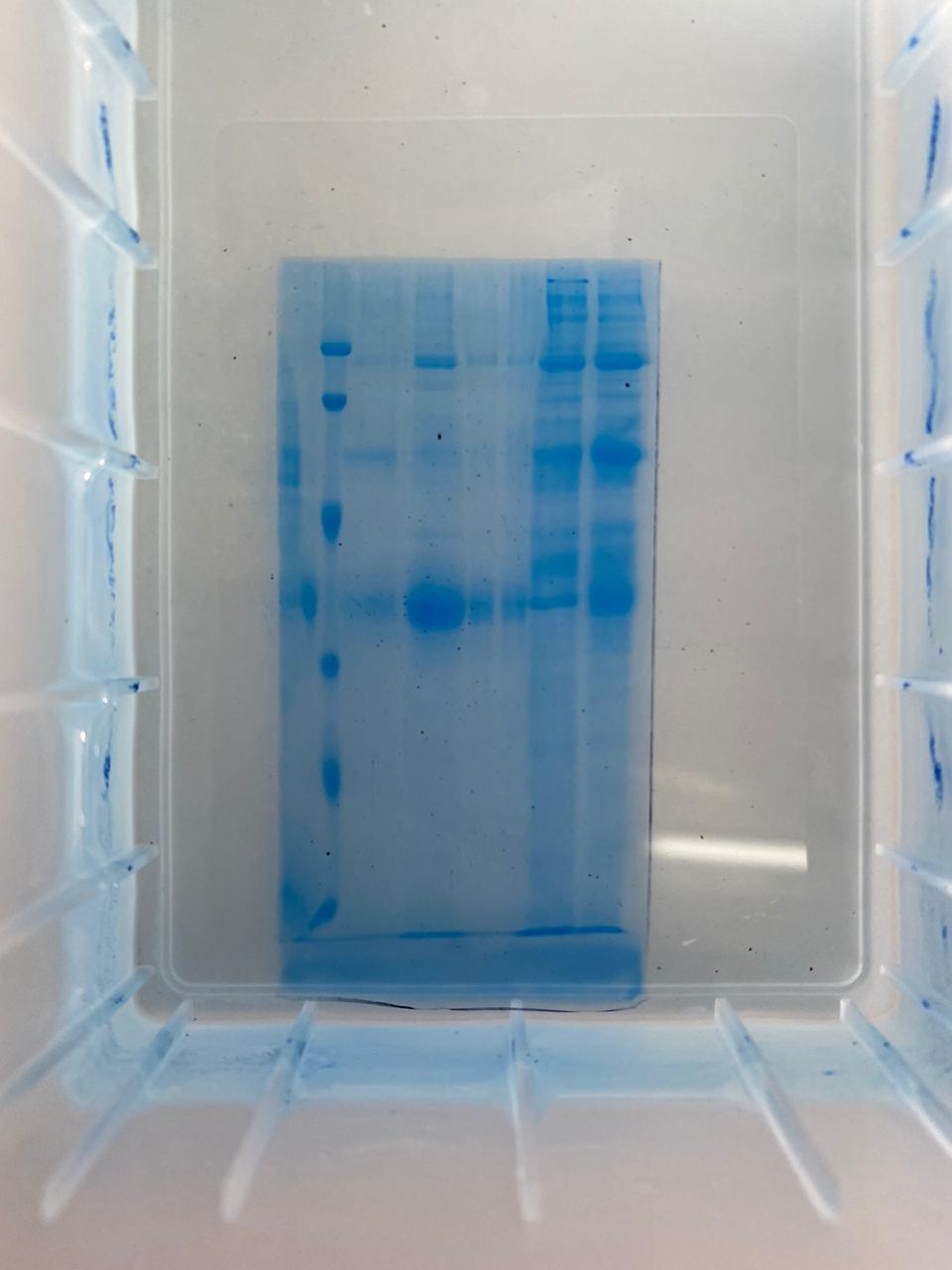


**X**

Fig _1E. e4c3a97b-5a15-442f-a852-0a600ea1cc47.jpg

17 august 2020
RBDv1 SDS-PAGE 12.5%, purification on nitrilotriacetic acid (NTA)
lane 1: harvested medium, 5 uL concentrate
lane 2: non-binding fraction, 5 uL concentrate
lane 3: 50 mM imidazole elution, 5 uL concentrate
lane 4: 50 mM imidazole elution (tail), 5 uL concentrate
lane 5: 250 mM imidazole elution, 5 uL concentrate
lane 6: Na-EDTA elution, 5 uL concentrate
lane 7: Prestained Protein Molecular Weight Marker #26612, 5 uL
lane 8: 250 mM imidazole elution, 0.5 uL concentrate – non-relevant data

All samples are 20x concentrates through Vivaspin PES membrane 10000 MWCO (Sartorius, UK, VS0102), SDS-PAGE 12.5% in reducing conditions (50 mM DTT)


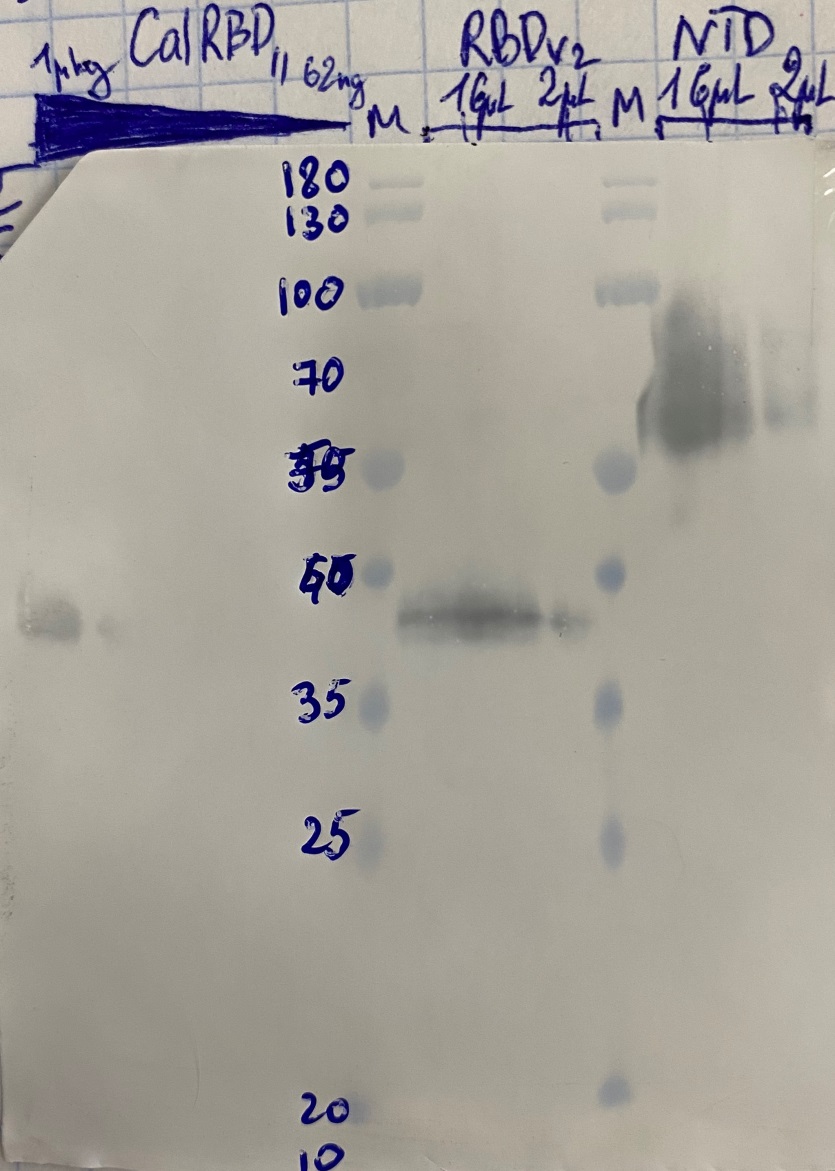


**X X X X X X X X**

Fig 2_B - CoV_WB_20200908_RBDv2_NTD_2uM.jpg

17 august 2020
RBDv1 blot
lanes 1-5: RBDv1 purified calibrator, batch 11, 1 ug to 62 ng per well
lane 6: PageRuler Prestained Protein Ladder #26616, 3 uL
lane 7: 2 uM RBDv2 culture, 10 uL concentrate
lane 8: 2 uM RBDv2 culture, 1 uL concentrate – non-relevant data
lane 9: PageRuler Prestained Protein Ladder #26616, 3 uL
lane 10: 2 uM NTD culture, 10 uL concentrate – non-relevant data
lane 11: 2 uM NTD culture, 1 uL concentrate – non-relevant data

RBDv2 and NTD samples are 20x concentrates through Vivaspin PES membrane 10000 MWCO (Sartorius, UK, VS0102), SDS-PAGE 12.5% in reducing conditions (50 mM DTT)


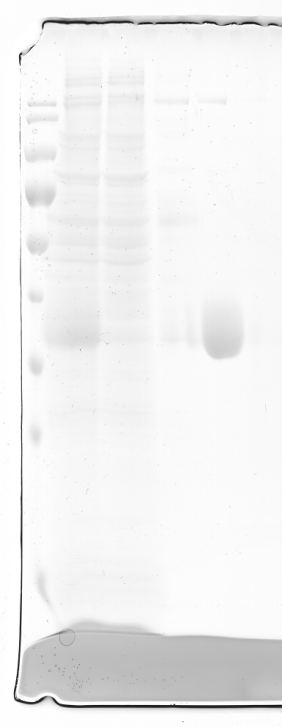


Fig 2_C - CoV_SDS_PAGE_20201002_RBDv2_PR03.tif

28 september 2020
RBDv2 SDS-PAGE 12.5%, 1L flask, 2 uM culture, purification on nitrilotriacetic acid (NTA), batch 03
lane 1: PageRuler Prestained Protein Ladder #26616, 5 uL
lane 2: harvested medium, 10 uL
lane 3: non-binding fraction, 10 uL
lane 4: 50 mM imidazole, 4 uL
lane 5: 250 mM imidazole elution, 2 uL
lane 6: Na-EDTA elution, 4 uL

Samples were loaded without concentration, the loaded volumes were proportional to the volumes of unconcentrated fractions. SDS-PAGE 12.5% in reducing conditions (50 mM DTT)


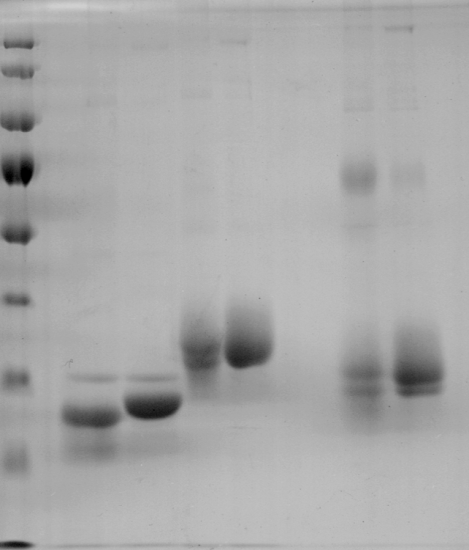

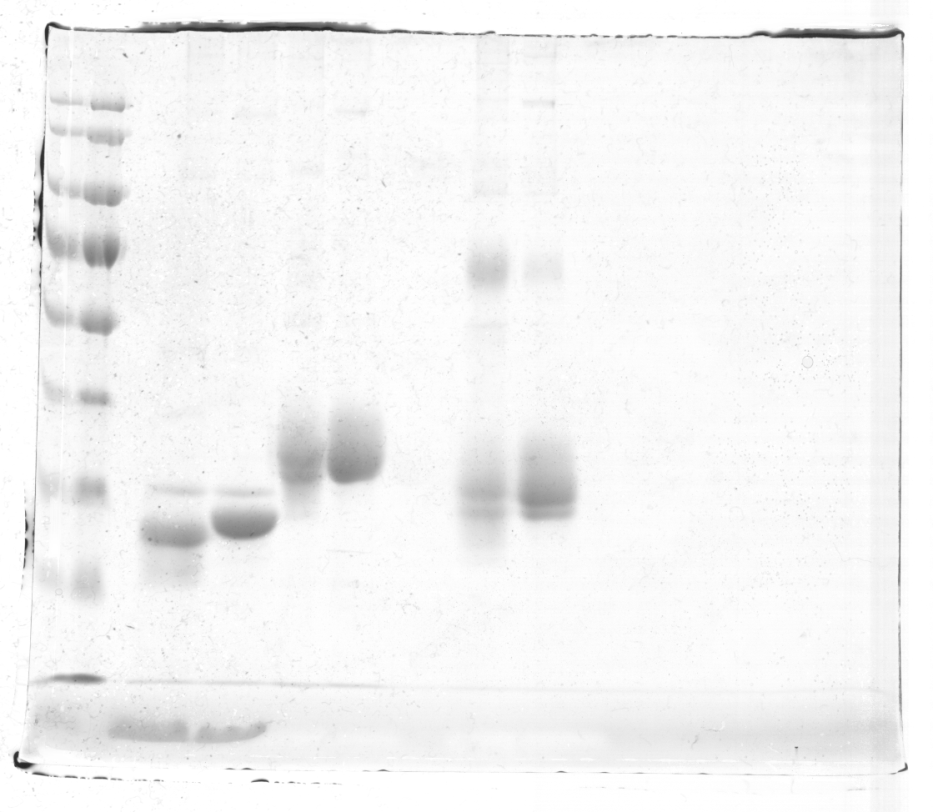


Fig 4_A - CoV_SDS_PAGE_20201014_RBD.tif

14 october 2020
RBDv1 vs RBDv2 SDS-PAGE 12.5%, PNGase treatment
lane 1: PageRuler Prestained Protein Ladder #26616, 5 uL
lane 2: X
lane 3: RBDv1, +PNGase F, +DTT , 5 ug
lane 4: RBDv2, +PNGase F, +DTT , 5 ug
lane 5: RBDv1, -PNGase F, +DTT , 5 ug
lane 6: RBDv2, -PNGase F, +DTT , 5 ug
lane 7: X
lane 8: X
lane 9: RBDv1, -PNGase F, -DTT , 5 ug
lane 10: RBDv2, -PNGase F, -DTT , 5 ug

For all samples volumes were adjusted to the final volume of 15 uL with 4x DualColor loading buffer (Fermentas, #R1011)
